# Supplementary material for: Liquid crystalline inverted lipid phases encapsulating siRNA enhance lipid nanoparticle mediated transfection
Source: Nat Commun. 2024 Feb 12;15:1303. doi: 10.1038/s41467-024-45666-5 (PMC10861598; doi:10.1038/s41467-024-45666-5)
Supplement: Supplementary file 1 — Supplementary Information [file 41467_2024_45666_MOESM1_ESM.pdf]

Liquid crystalline Inverted Lipid Phases Encapsulating siRNA Enhance Lipid Nanoparticle Mediated Transfection

Roy Pattipeiluhu[1,2,4], Ye Zeng[1], Marco M.R.M. Hendrix[3], Ilja K. Voets[3], Alexander Kros[1] and Thomas H. Sharp[2]

[1]Supramolecular and Biomaterials Chemistry, Leiden Institute of Chemistry, Leiden University, Einsteinweg 55, 2333CC Leiden, The Netherlands

[2]Department of Cell and Chemical Biology, Leiden University Medical Center, Einthovenweg 20, 2333ZC Leiden, The Netherlands

[3]Self-Organizing Soft Matter, Department of Chemical Engineering and Chemistry, Eindhoven University of Technology, De Wielen 10, 5612AZ Eindhoven, The Netherlands

[4]Present address: BioNTech SE, An der Goldgrube 12, 55131, Mainz, Germany

Table of Contents

Supplementary Figures: 4
Supplementary Figure 1. LNP components used in this study. 4
Supplementary Figure 2. Schematic of LNP assembly. 4
Supplementary Figure 3. Additional cryoTEM images of 10PE-LNP-NP1, 30-PE-LNP-NP1 and 49PE-LNP-NP1. 5
Supplementary Figure 4. CryoTEM images of 10PE-LNP-NP6 and 10PE-LNP-noRNA. 6
Supplementary Figure 5. Encapsulation efficiency (%) of all formulations formulated at NP ratios of 6 and 1. 7
Supplementary Figure 6. CryoTEM images of 30PE-LNP-NP6 and 30PE-LNP-noRNA. 8
Supplementary Figure 7. CryoTEM images of 49PE-LNP-NP6 and 49PE-LNP-noRNA. 9
Supplementary Figure 8. Co-existence of lamellar, straight line and hexagonal structures in 30PE-LNP-NP1. 10
Supplementary Figure 9. Correlation of filled liquid crystalline inverse hexagonal phases with LNP particle size. 10
Supplementary Figure 10. Comparison of identified structures in cryoTEM of 49PE-LNP to SAXS profiles 11
Supplementary Figure 11. CryoET slices of an individual 49PE-LNP-noRNA particle. 12
Supplementary Figure 12. SAXS profiles after incubation at 37 °C for 7-12 hours. 12
Supplementary Figure 13. CryoTEM images of 49PE-LNP-NP1 containing 0.1 mol% of DiD. 13
Supplementary Figure 14. Cell viability study of cell lines treated with LNPs........ 14
Supplementary Figure 15. Cellular transfection HeLa cells lines treated with 49PE-LNP-NP1 ± 0.1 mol% DiD or negative control siRNA. 15
Supplementary Figure 16. CryoTEM images of anionic LUVs. 15
Supplementary Figure 17. Interaction of 10PE-DS-LNP-NP1 with anionic LUVs.. 17
Supplementary Figure 18. Interaction of 30PE-LNP-NP1 with anionic LUVs........ 18
Supplementary Figure 19. Additional cryoTEM images of 10PE-LNP-NP1 interaction with anionic LUVs 19
Supplementary Figure 20. Additional cryoTEM images of 49PE-LNP-NP1 interaction with anionic LUVs 20
Supplementary Tables: 21
Supplementary Table 1. siRNA sequences used in this study. 21
Supplementary Table 2. Dynamic Light Scattering (DLS) and ζ-potential data..... 21

Supplementary Table 3. IC_{50}-values of LNPs determined from eGFP silencing in U2OS and HeLa cell lines. 22

Supplementary Table 4. Comparison of hexagonal and cubic model calculations based on Braggs peak maxima.......... 23

References 23

Supplementary Figures:

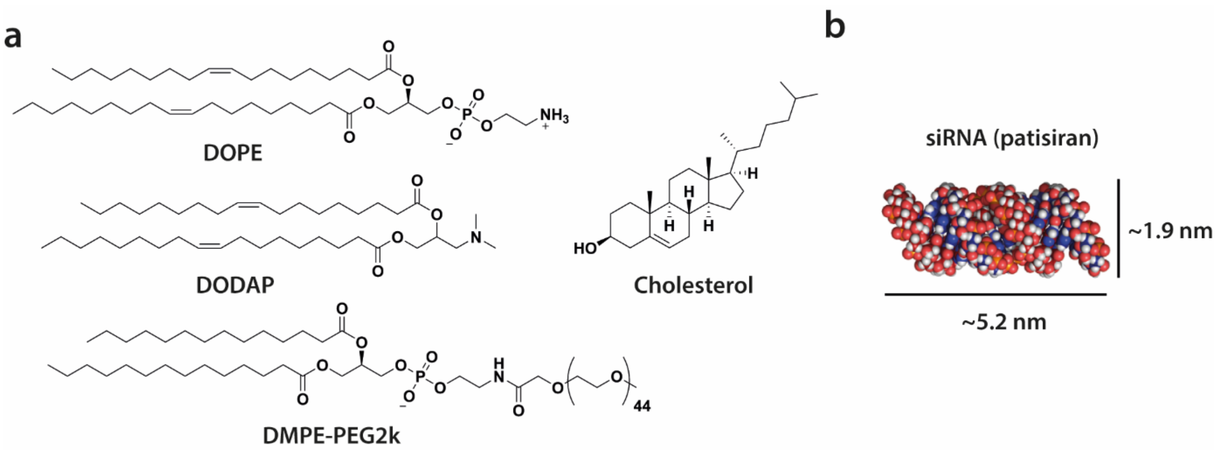

Supplementary Figure 1. LNP components used in this study.

(**a**) Chemical structures of lipid components used in LNP assembly. (**b**) RNA A-form structure of Patisiran®, model was created using UCSF Chimera. Width and length were determined in PyMol.

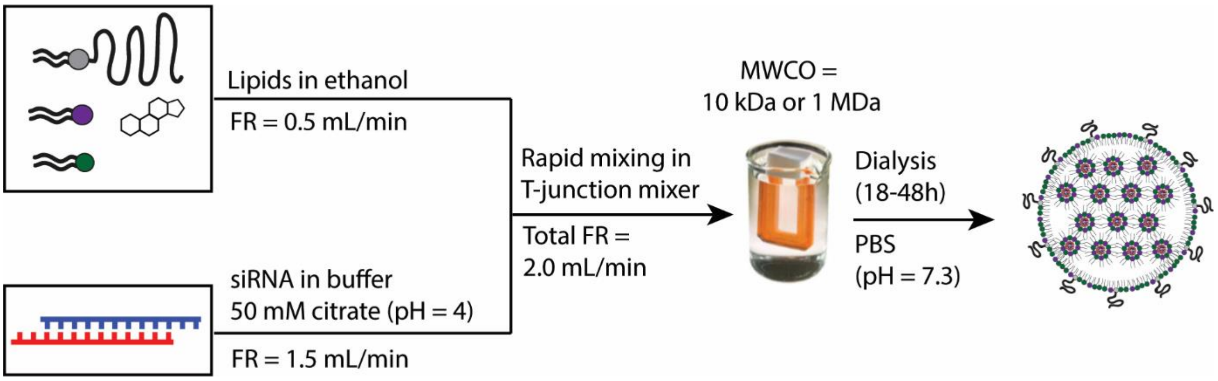

Supplementary Figure 2. Schematic of LNP assembly.

Lipids in ethanol were mixed through a T-junction mixture with siRNA in 50 mM citrate buffer (pH = 4.0) at respective flow rates (FRs) of 0.5 mL/min and 1.5 mL/min. The acquired suspension was dialyzed against PBS to obtain the fully assembled LNPs. Abbreviations used: FR = flow rate, MWCO = molecular weight cut-off, PBS = phosphate buffered saline.

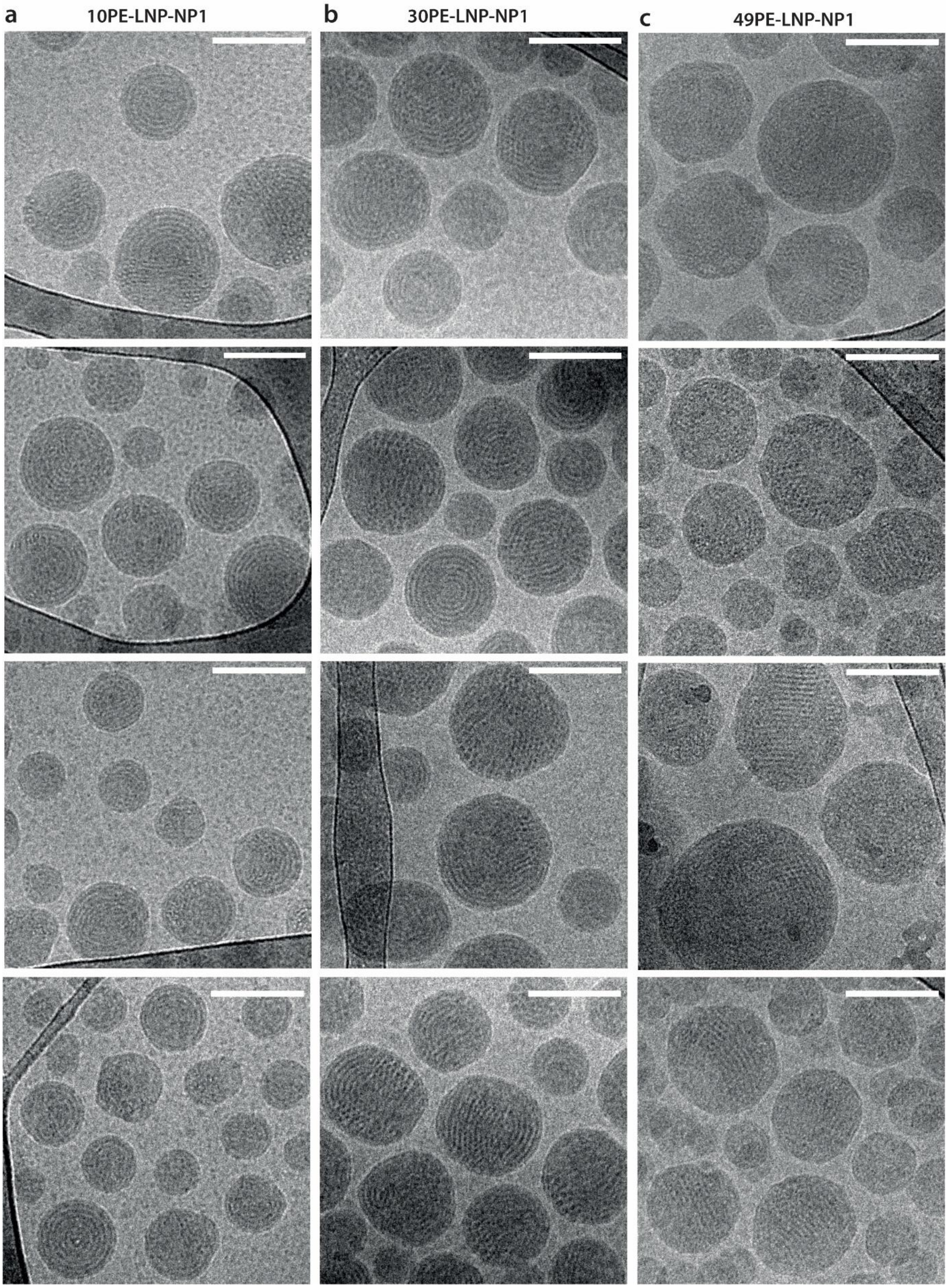

Supplementary Figure 3. Additional cryoTEM images of 10PE-LNP-NP1, 30-PE-LNP-NP1 and 49PE-LNP-NP1.

(**a-c**) Imaging was performed on a 120 kV Tecnai T12 as described in the Materials and Methods section. All scale bars are 100 nm.

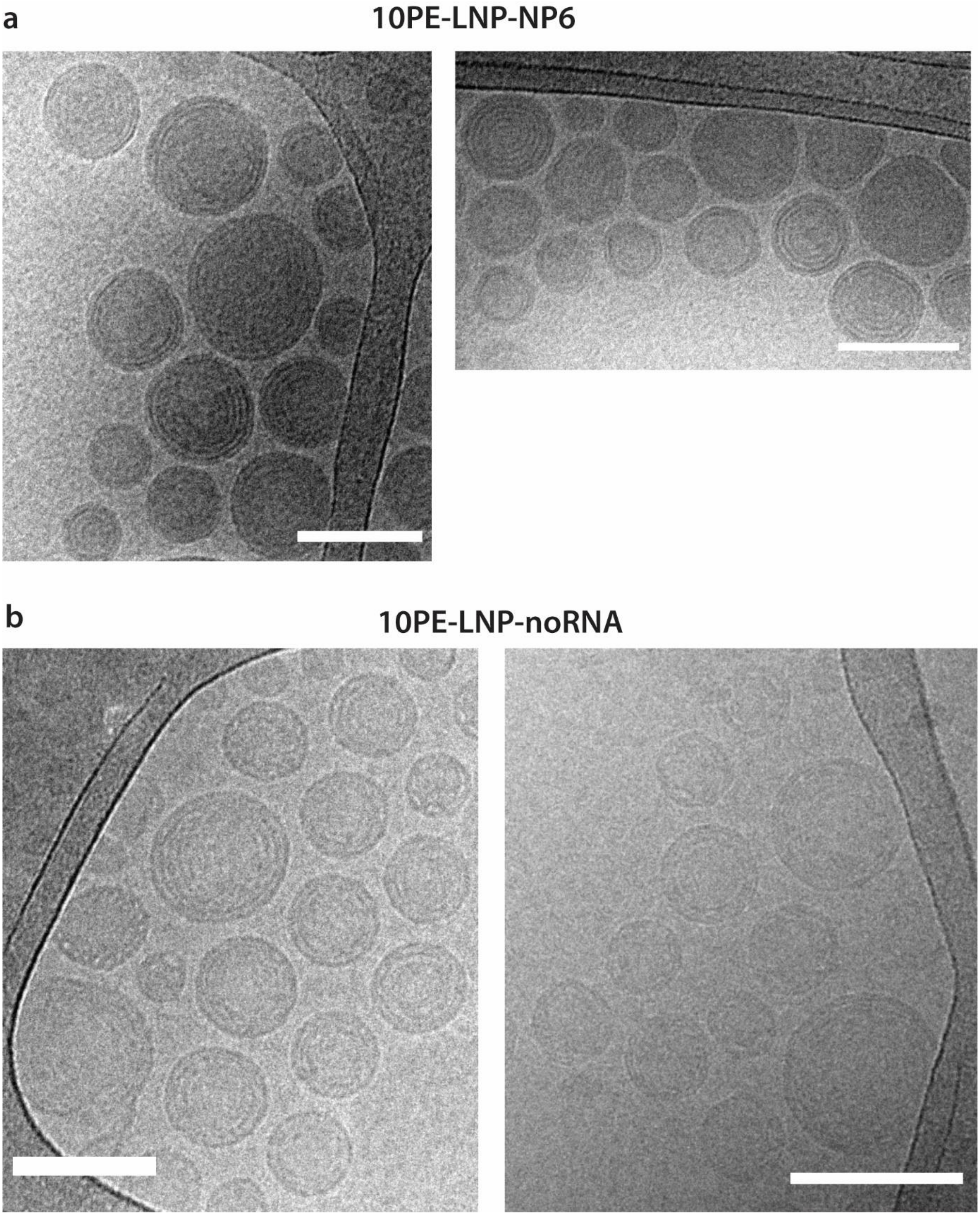

Supplementary Figure 4. CryoTEM images of 10PE-LNP-NP6 and 10PE-LNP-noRNA.

(**a-b**) Imaging was performed on a 120 kV Tecnai T12 as described in the Materials and Methods section. All scale bars are 100 nm.

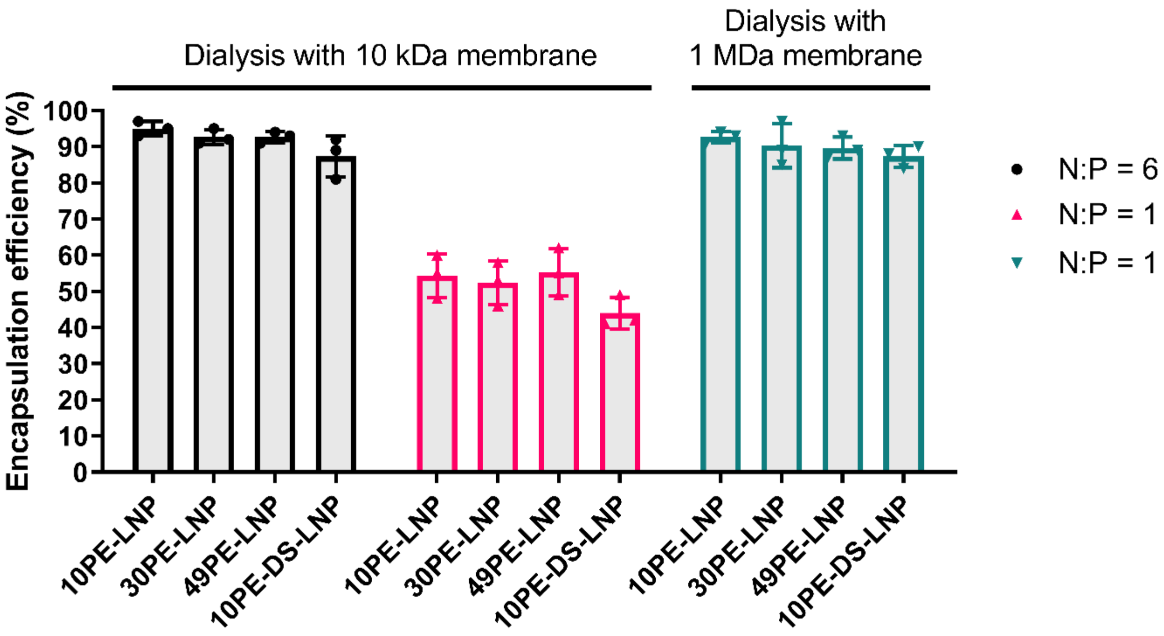

Supplementary Figure 5. Encapsulation efficiency (%) of all formulations formulated at NP ratios of 6 and 1.

For the NP ratio of 1, dialysis performed with 1 MDa membranes (Spectra-Por® Float-A-Lyzer® G2, Thermo Scientific) shows the efficient removal of non-encapsulated siRNA over dialysis with 10 kDa membranes (Slide-A-Lyzer™, Thermo Scientific). Dialysis time was 48 hours in all cases. Bar plots and error bars represent the average and standard deviation from a triplicate of independent assemblies.

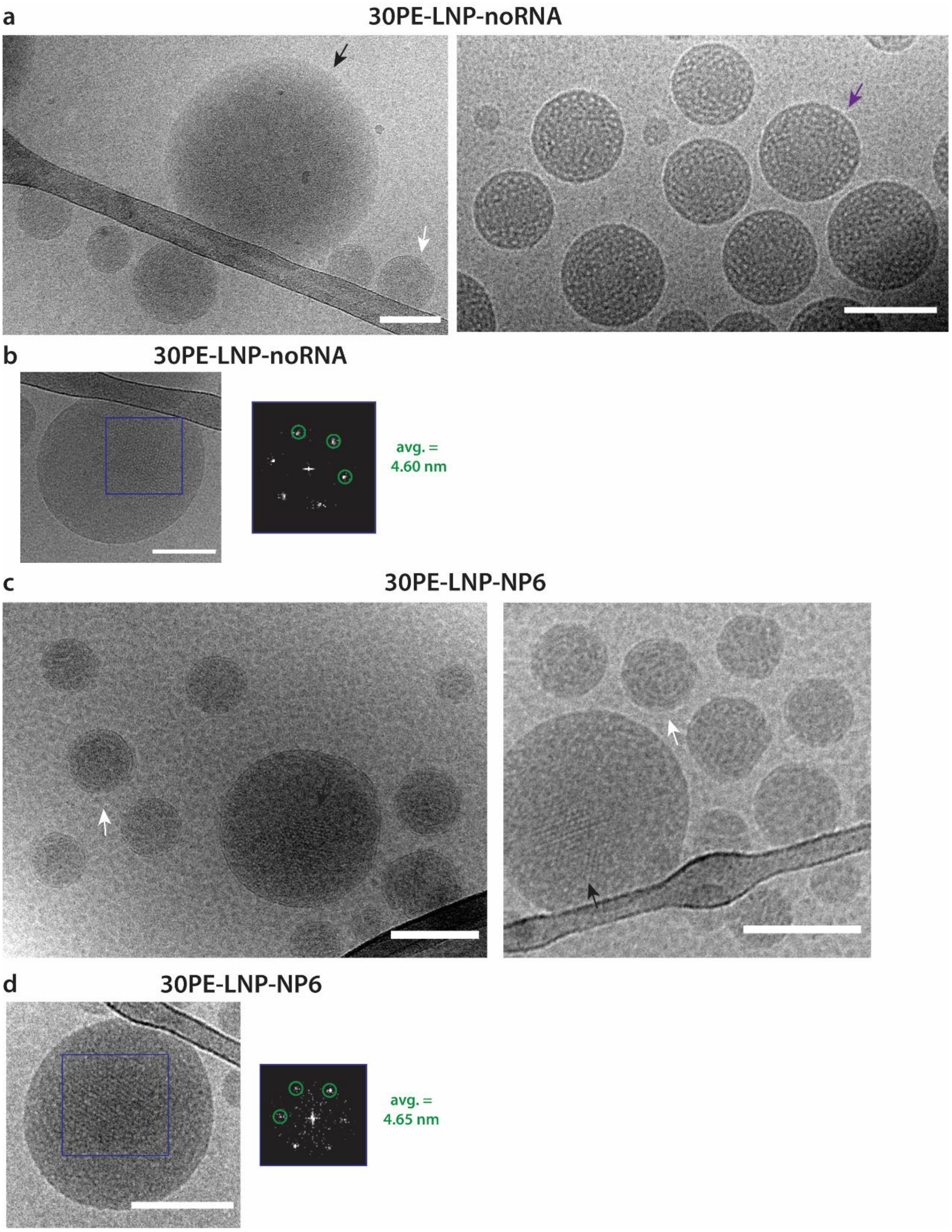

Supplementary Figure 6. CryoTEM images of 30PE-LNP-NP6 and 30PE-LNP-noRNA.

(**a-d**) Imaging was performed on a 120 kV Tecnai T12 as described in the Materials and Methods section. All scale bars are 100 nm. Black arrows indicate the presence of tubular inverse hexagonal structures. White arrows indicate the presence of concentric lamellar structures. Purple arrows indicate the presence of undefined lipid structures. FFT values represent the average of the [001] structure of the selected areas in individual particles.

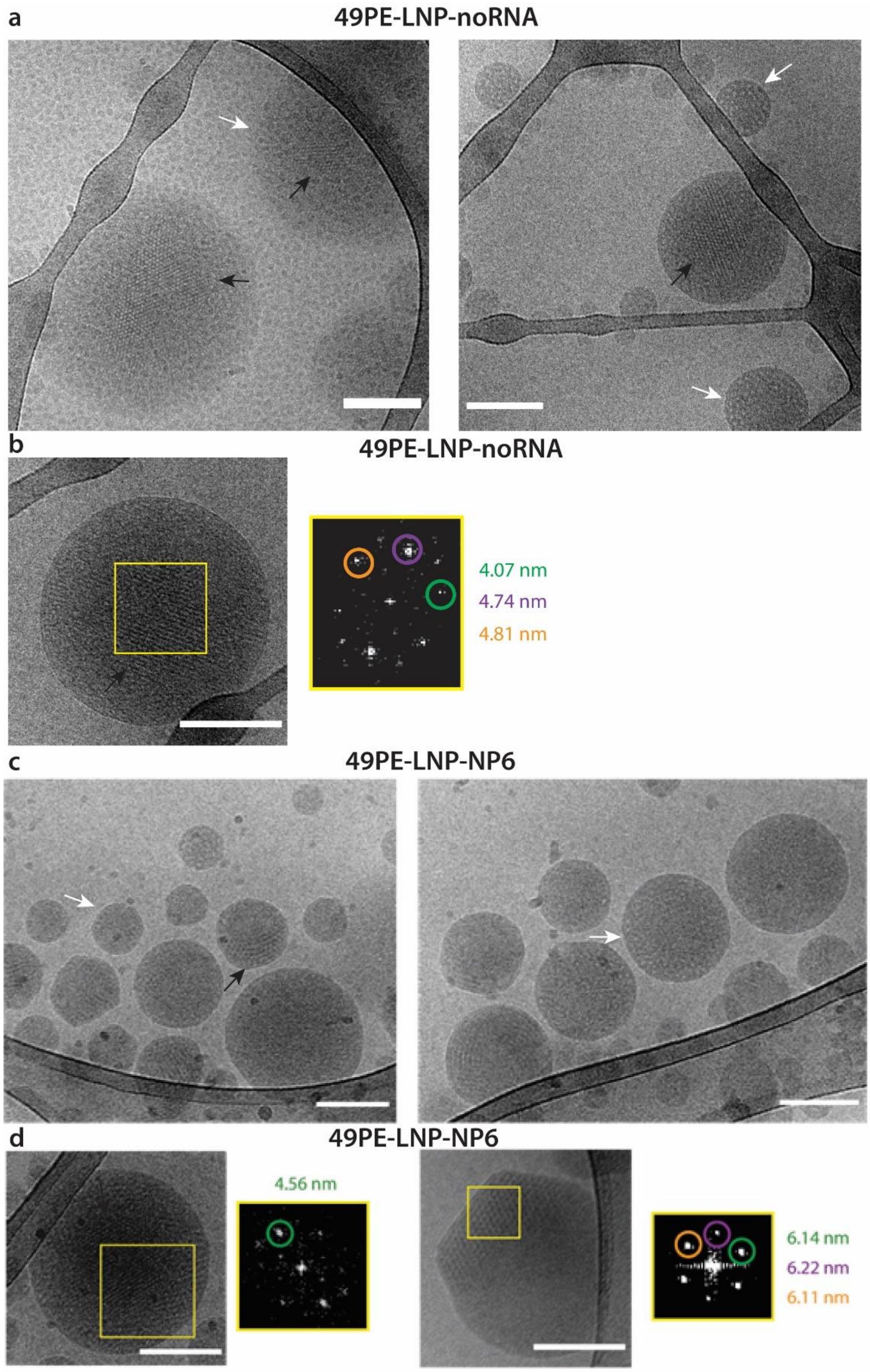

Supplementary Figure 7. CryoTEM images of 49PE-LNP-NP6 and 49PE-LNP-noRNA.

(**a-d**) Imaging was performed on a 120 kV Tecnai T12 as described in the Materials and Methods section. All scale bars are 100 nm. FFT values are matched by color in the selected areas in individual particles. Black arrows indicate the presence inverse hexagonal structures throughout the LNP core. White arrows indicate the presence of spherical structures described in Supplementary Figure 10.

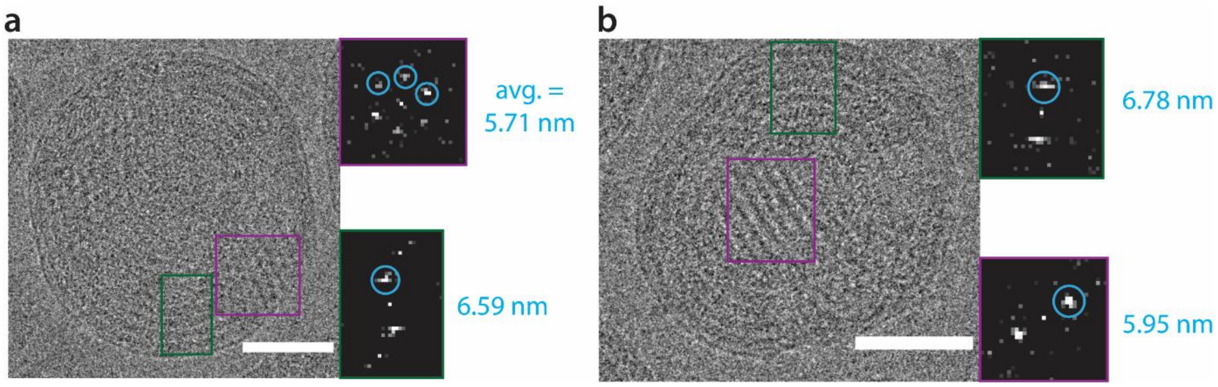

Supplementary Figure 8. Co-existence of lamellar, straight line and hexagonal structures in 30PE-LNP-NP1.

(**a,b**) cryoTEM images of 30PE-LNP-NP1 particles, showing the co-existence of structures within the same particle, along with FFT analysis of the color coded selections. Purple selections represent hexagonal or straight line structures of inverse hexagonal structures, green selections represent lamellar structures. Imaging was performed on a 300 kV Titan Krios 2 as described in the Materials and Methods section. All scale bars are 50 nm.

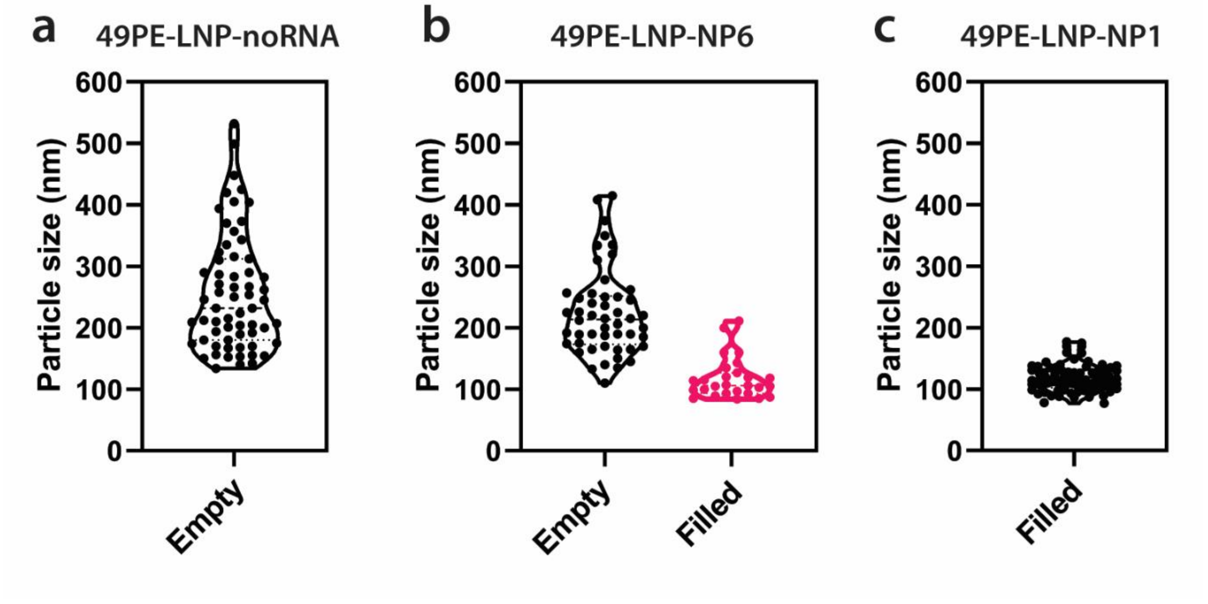

Supplementary Figure 9. Correlation of filled liquid crystalline inverse hexagonal phases with LNP particle size.

Relation between lattice spacings show in **Figure 3b** to particle size for 49-LNP variants at different siRNA amounts. (**a**) 49PE-LNP-noRNA (n = 63, 257 nm ± 95 nm), (**b**) 49PE-LNP-NP6, empty (n = 50, 224 nm ± 70 nm) NP = 6 filled (n = 27, 117 nm ± 33 nm) and (**c**) 49PE-LNP-NP1 (n = 81, 118 nm ± 22 nm).

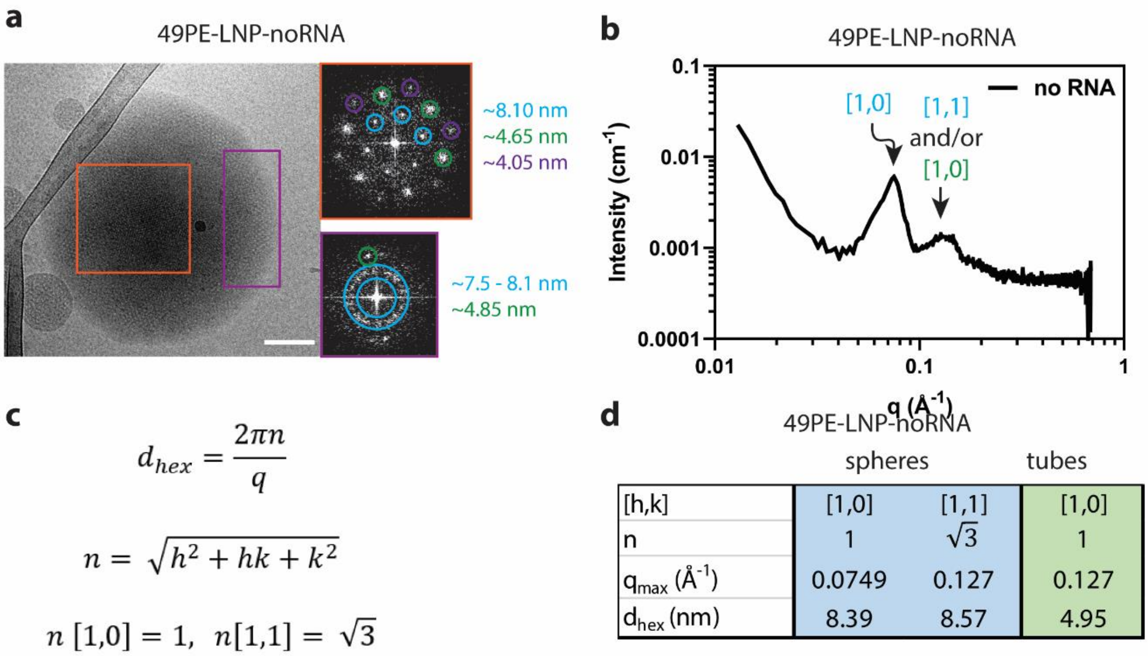

c

$$d_{hex} = \frac{2\pi n}{q}$$

$$n = \sqrt{h^2 + hk + k^2}$$

$$n\,[1{,}0] = 1,\;\; n[1{,}1] = \sqrt{3}$$

d

49PE-LNP-noRNA

	spheres		tubes
[h,k]	[1,0]	[1,1]	[1,0]
n	1	$\sqrt{3}$	1
q_{max} (Å^{-1})	0.0749	0.127	0.127
d_{hex} (nm)	8.39	8.57	4.95

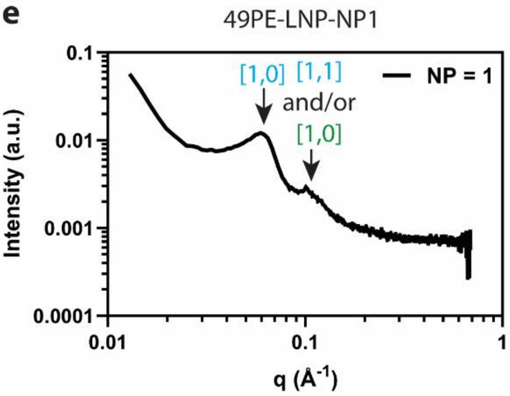

f

49PE-LNP-NP1

	polymorphic		tubes
[h,k]	[1,0]	[1,1]	[1,0]
n	1	$\sqrt{3}$	1
q_{max} (Å^{-1})	0.0590	0.101	0.101
d_{hex} (nm)	10.65	10.78	6.22

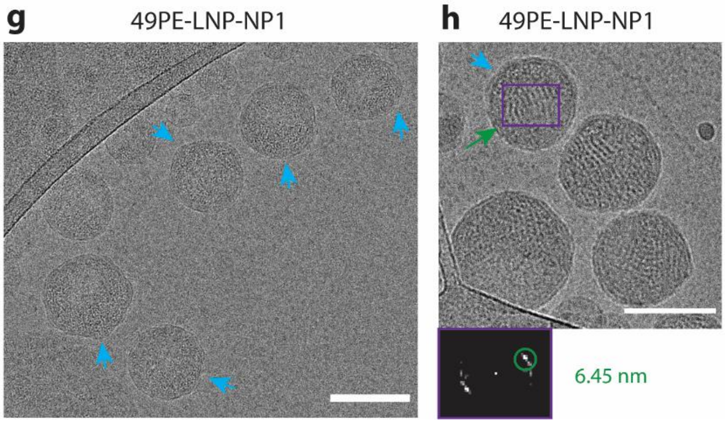

Supplementary Figure 10. Comparison of identified structures in cryoTEM of 49PE-LNP to SAXS profiles

(a) CryoTEM image of large 49PE-LNP-noRNA particle showing both hexagonally packed spheres and inverse hexagonal tubular structures. (b) SAXS profile of 49PE-LNP-noRNA derived from **Figure 1f**, with indications of Bragg peaks with Miller indices. (c) Formulas used for the calculation of the d-spacing assuming a hexagonally packed structure. (d) d-spacing calculations for 49PE-LNP-noRNA (e) SAXS profile of 49PE-LNP-NP1 derived from **Figure 1f**, with indications of Bragg peaks with Miller indices. (f) Formulas used for the calculation of the d-spacing assuming a hexagonally packed structure. (g-h)

cryoTEM images of 49PE-LNP-NP1 showing polymorphic (blue arrows) and inverse hexagonal tubular structures (green arrow).

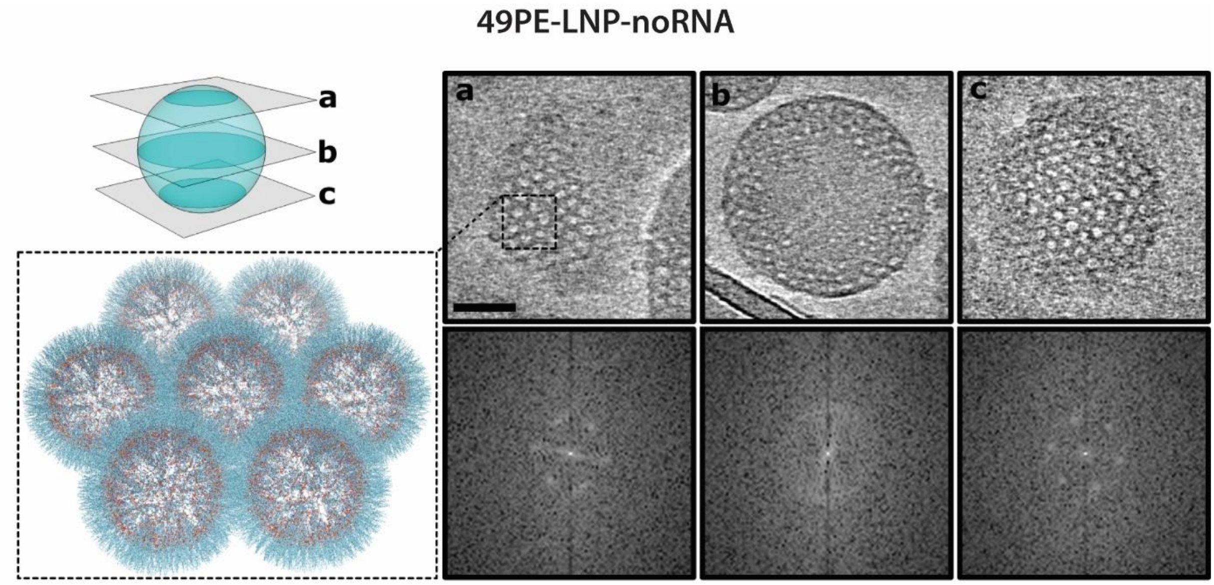

Supplementary Figure 11. CryoET slices of an individual 49PE-LNP-noRNA particle.

Tomographic slices through an individual 49PE-LNP-noRNA particle (cyan) at the heights indicated with (**a,b,c**), revealing the hexagonally packed spheres through the LNP, with an amorphous core. Model displays spheres with lipid tails pointed outwards and an average size of ~8 nm. Scale bar = 50 nm.

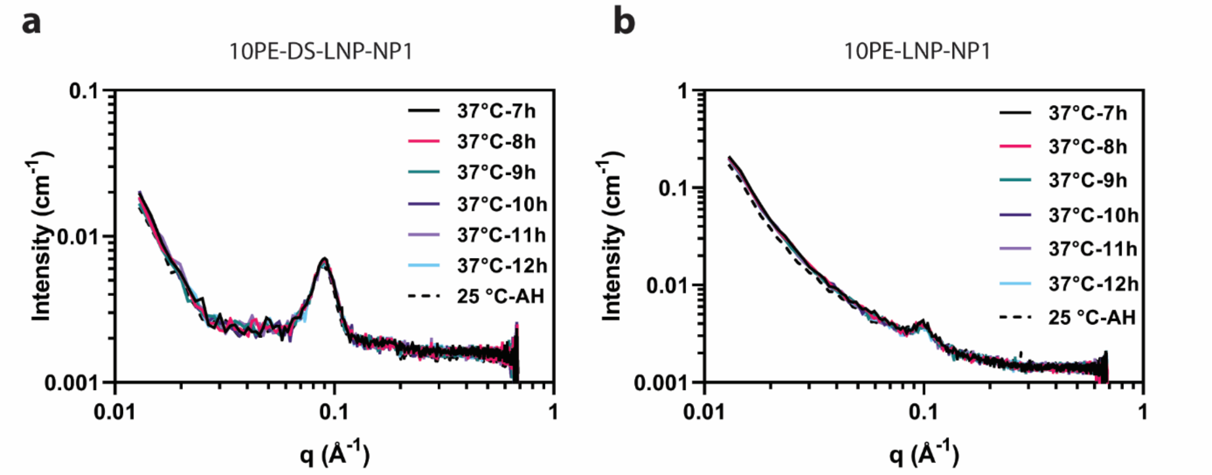

Supplementary Figure 12. SAXS profiles after incubation at 37 °C for 7-12 hours.

SAXS profiles at additional points of incubation at 37 °C and 25 °C after heating (AH) for (**a**) 10PE-DS-LNP-NP1 and (**b**) 10PE-LNP-NP1.

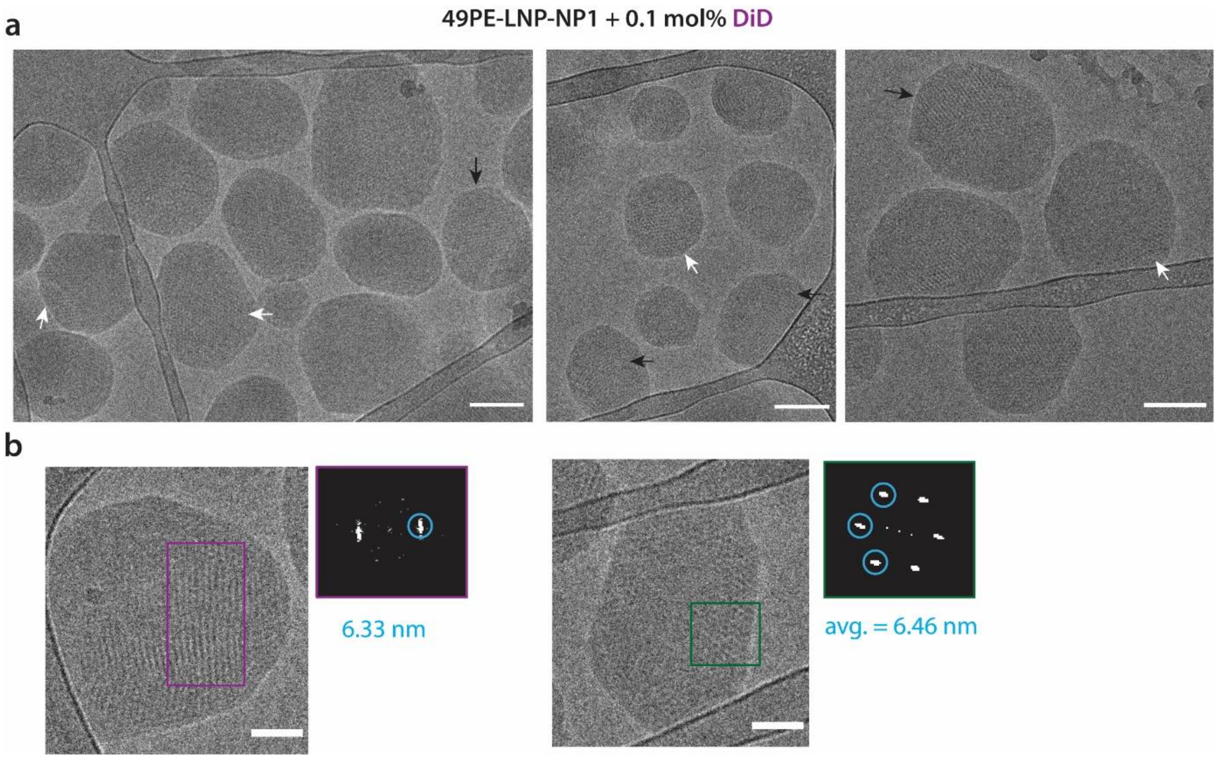

Supplementary Figure 13. CryoTEM images of 49PE-LNP-NP1 containing 0.1 mol% of DiD.

(**a,b**) Imaging was performed on a 120 kV Tecnai T12 as described in the Materials and Methods section. Black arrows indicate the presence of straight lines coming from present inverse hexagonal structures. White arrows indicate the presence of inverse hexagonal structures. Scale bars in **a** are 100 nm, scale bars in **b** are 50 nm.

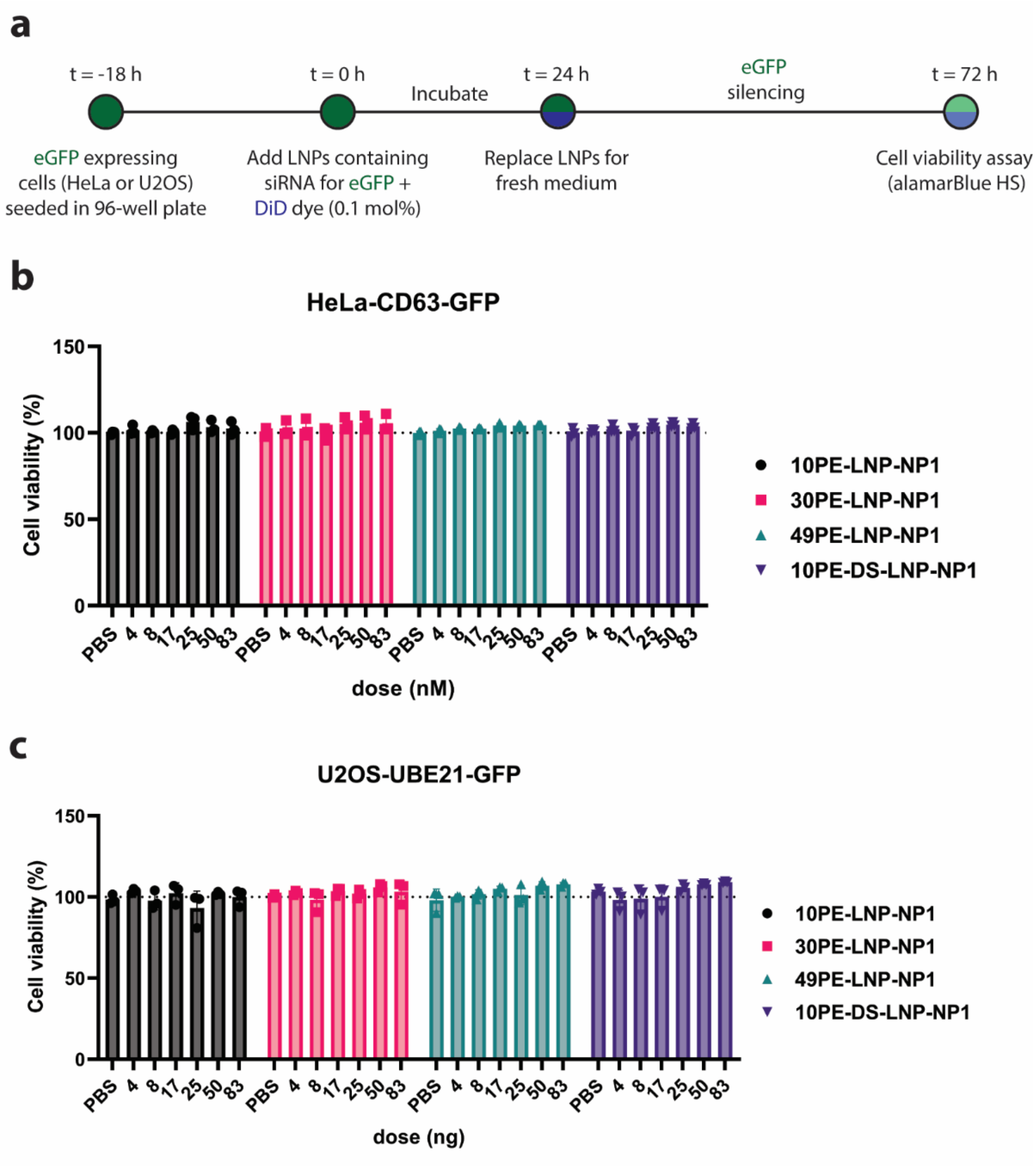

Supplementary Figure 14. Cell viability study of cell lines treated with LNPs.

(**a**) Schematic showing the timeline of the cell viability study conducted. (**b-c**) Results of the cell-viability assay (alamarBlue HS) of the HeLa and U2OS cell lines. n = 3.

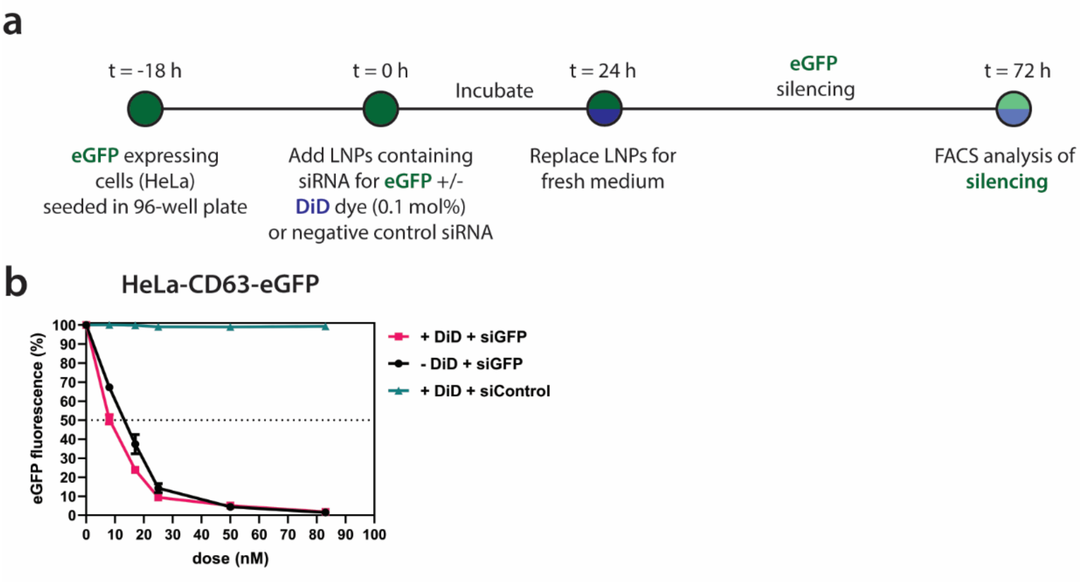

Supplementary Figure 15. Cellular transfection HeLa cells lines treated with 49PE-LNP-NP1 ± 0.1 mol% DiD or negative control siRNA.

(**a**) Schematic showing the timeline of eGFP silencing experiment with control LNP formulations. (**b**) Results of the eGFP fluorescence silencing of control formulations as a function of dose in HeLa-CD63-eGFP cells. n = 3.

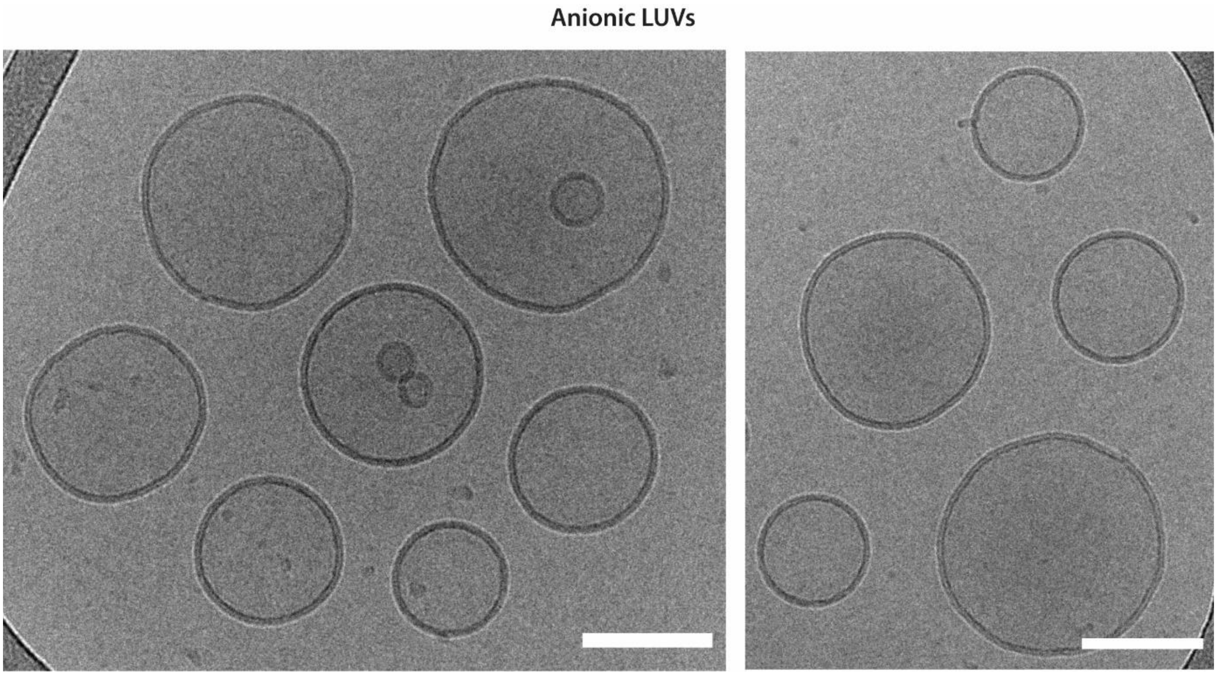

Supplementary Figure 16. CryoTEM images of anionic LUVs.

LUVs are composed of PC:PE:PS:Chol:PI at a ratio of 50:27:10:10:3 mol%. Sample vitrification in the mixture of 100 mM citrate buffer and PBS (1:2 vol:vol) at 37 °C as described in the Materials and Methods section. All scale bars are 100 nm.

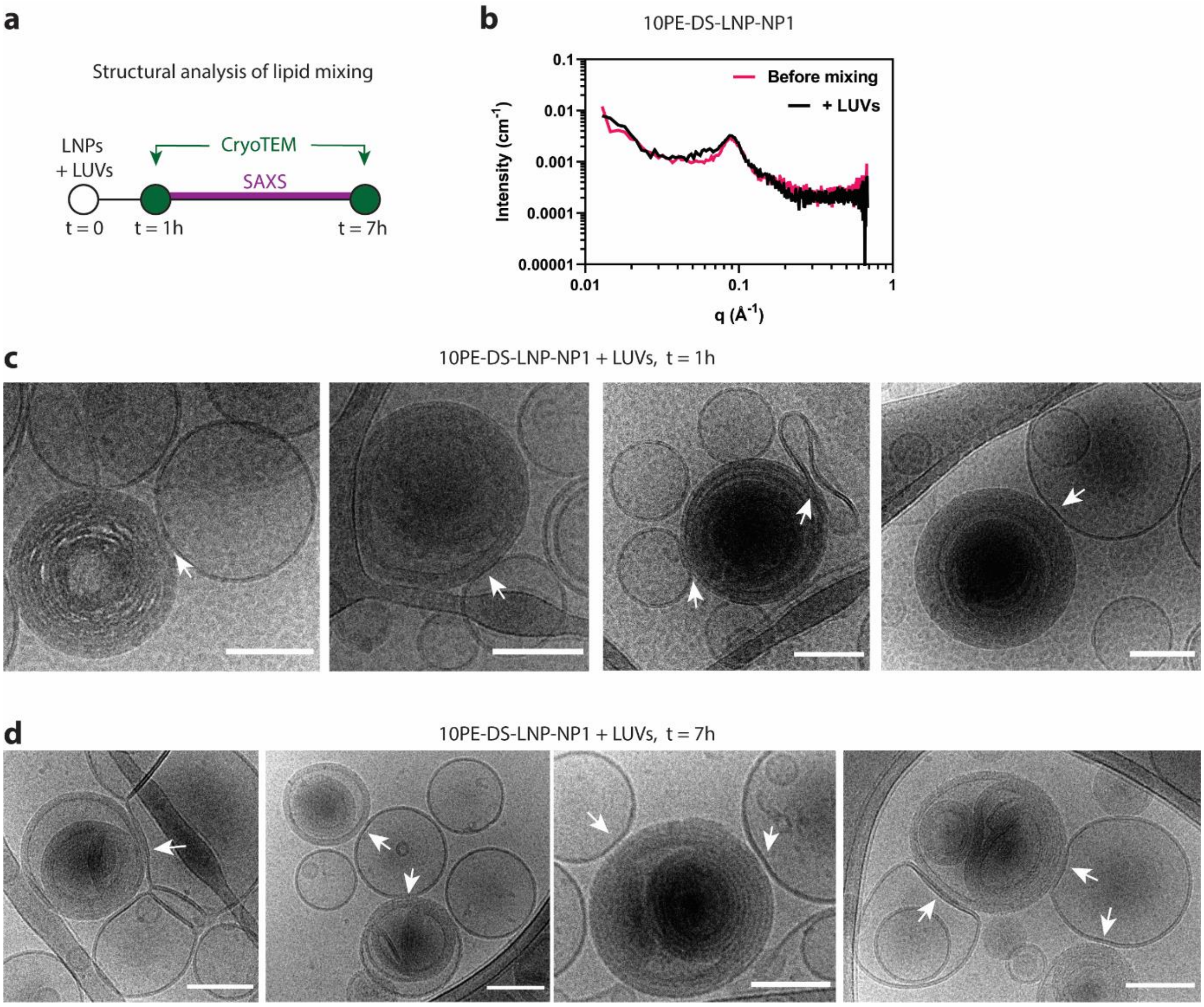

Supplementary Figure 17. Interaction of 10PE-DS-LNP-NP1 with anionic LUVs.

(**a**) Schematic representation depicting the experiments for the structural analysis of the LNP-LUV interaction. (**b**) SAXS profile of 10PE-DS-LNP-NP1 incubated with anionic LUVs. (**c,d**) CryoTEM images of 10PE-DS-LNP-NP1 incubated with anionic LUVs at pH 6 after 1 hour (t = 1h) and after 7 hours (t = 7h). White arrows indicate positions where docking between LNPs and LUVs is observed. All scale bars are 100 nm.

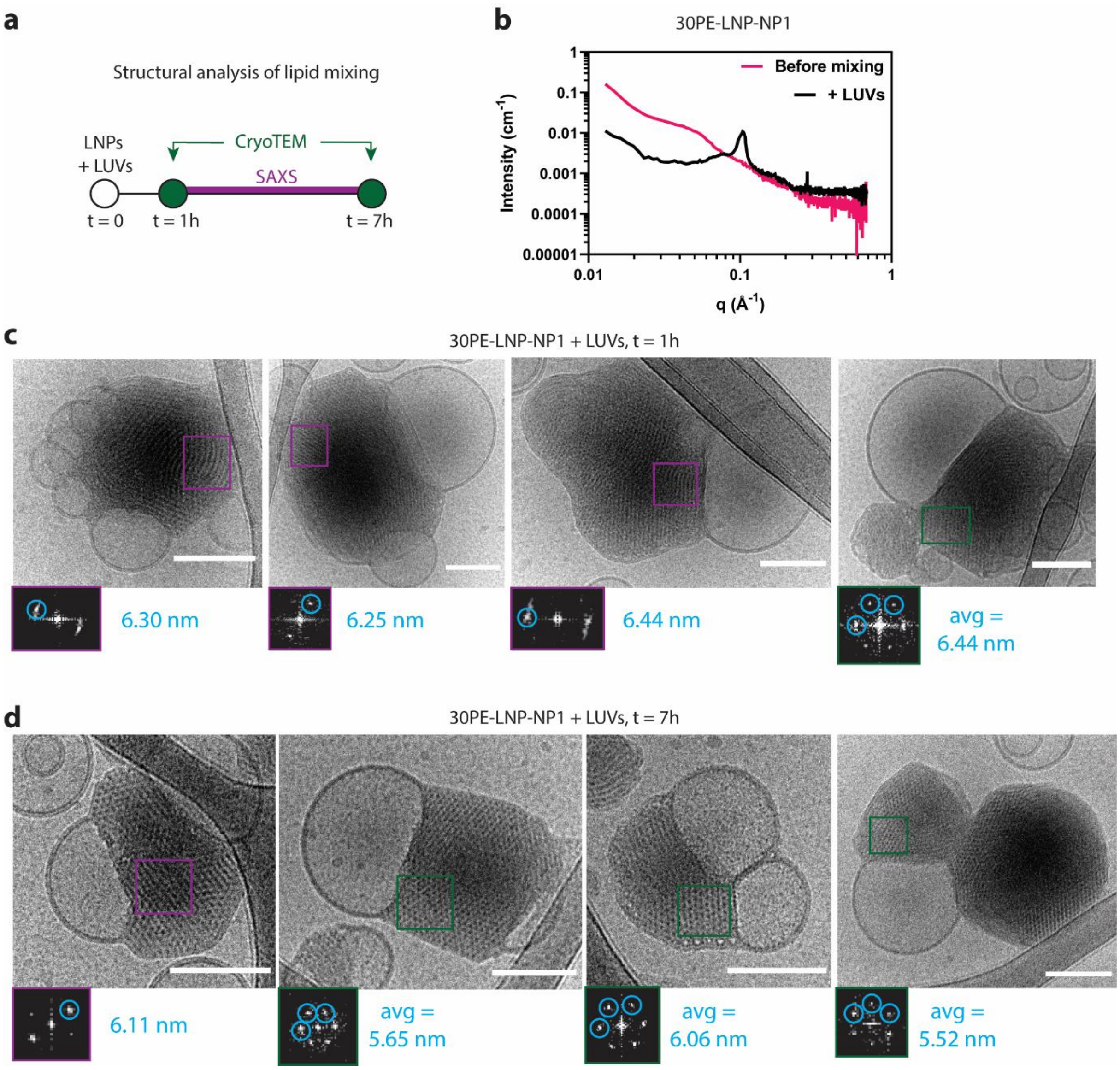

Supplementary Figure 18. Interaction of 30PE-LNP-NP1 with anionic LUVs

(**a**) Schematic representation depicting the experiments for the structural analysis of the LNP-LUV interaction. (**b**) SAXS profile of 30PE-LNP-NP1 incubated with anionic LUVs. (**c,d**) CryoTEM images and FFTs of selected areas of 30PE-LNP-NP1 incubated with anionic LUVs after 1 hour (t = 1h) and after 7 hours (t = 7h). All scale bars are 100 nm.

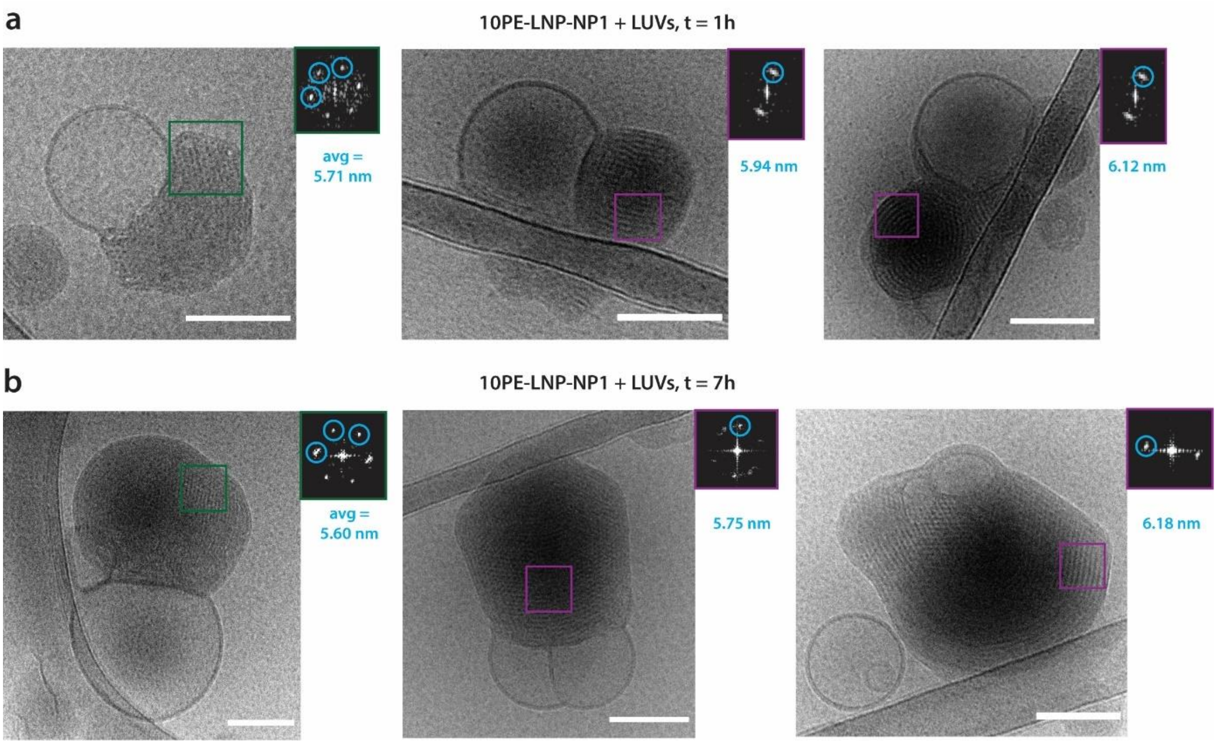

Supplementary Figure 19. Additional cryoTEM images of 10PE-LNP-NP1 interaction with anionic LUVs

(**a,b**) Additional CryoTEM images and FFTs of selected areas of 10PE-LNP-NP1 incubated with anionic LUVs after 1 hour (t = 1h) and after 7 hours (t = 7h). All scale bars are 100 nm.

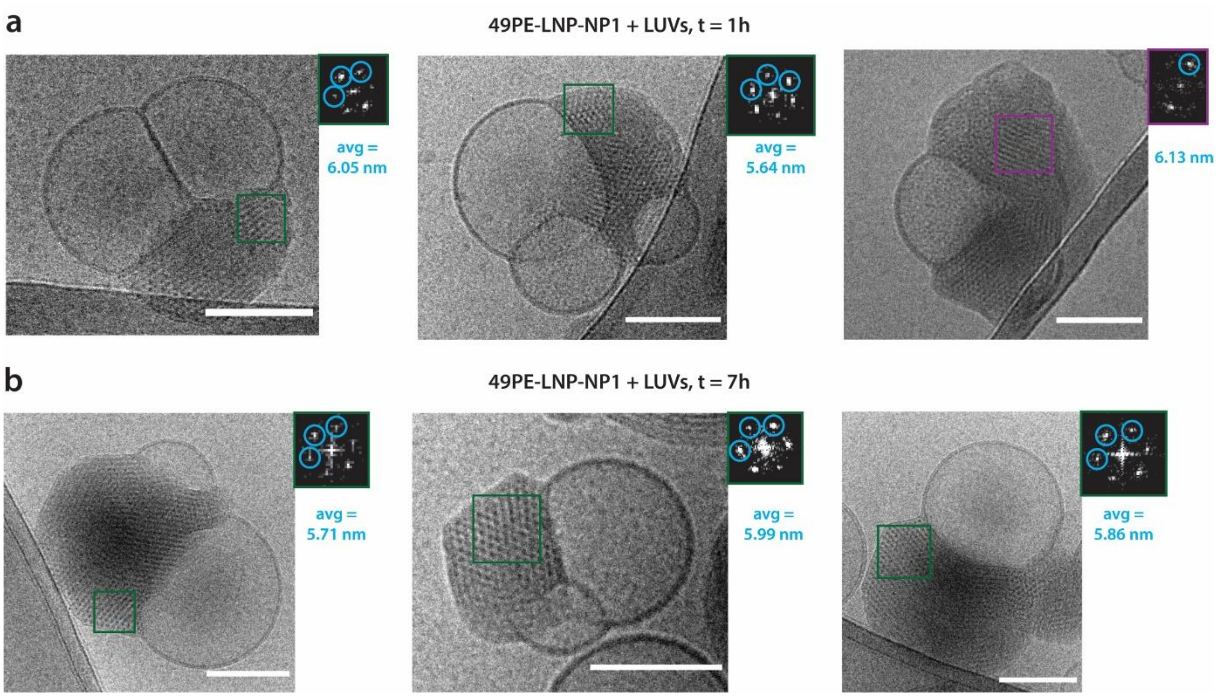

Supplementary Figure 20. Additional cryoTEM images of 49PE-LNP-NP1 interaction with anionic LUVs

(**a,b**) Additional CryoTEM images and FFTs of selected areas of 49PE-LNP-NP1 incubated with anionic LUVs after 1 hour (t = 1h) and after 7 hours (t = 7h). All scale bars are 100 nm.

Supplementary Tables:

Supplementary Table 1. siRNA sequences used in this study.

U_m and C_m are ribonucleotides with a 2'-OMe functionalization on the ribose ring. T_d, G_d and A_d are DNA ribonucleotides.

siRNA name	Sequence
Patisiran®	Sense: 5' $GU_mAAC_mC_mAAGAGU_mAU_mU_mC_mC_mAU_mT_dT_d$ 3'<br>Antisense: 3' $T_dT_dCAU_mUGGU_mU_mCUCAU_mAAGGU_mA$ 5'
siRNA-GFP	Sense: Phos-5'$ACCCUGAAGUUCAUCUGCACCACC_dG_d$ 3'<br>Antisense: 3'$CGGUGGUGCAGAUGAACUUCAGGGUCA_d$ 5'
negative control siRNA	Sense: 5'$CGUUAAUCGCGUAUAAUACGCGRUA_dT_d$ 3'<br>Antisense: 3'AUACGCGUAUUAUACGCGAUUAACGAC 5'

Supplementary Table 2. Dynamic Light Scattering (DLS) and ζ-potential data

Size and surface charge measurements of LNPs and LUVs. For measurements at 37 °C, samples were incubated for 1 hour prior to measurement.

LNP/LUV	RNA molecule	N/P ratio	Avg. size (nm) at 25 °C	PDI	ζ–potential (mV)	Avg. size (nm) at 37°C	PDI
10PE-LNP	Patisiran	1	115	0.089	-3.2	118	0.096
		6	125	0.102	-2.5	133	0.115
		no RNA	119	0.095	-4.5	121	0.099
	siRNA-eGFP	1	105	0.078	-1.6	106	0.080
30PE-LNP	Patisiran	1	132	0.096	-2.4	139	0.090
		6	129	0.110	-2.9	138	0.125
		no RNA	145	0.185	-2.7	152	0.200
	siRNA-eGFP	1	134	0.106	-4.5	138	0.111
49PE-LNP	Patisiran	1	144	0.096	-1.5	147	0.079
		6	149	0.111	-2.6	155	0.120
		no RNA	175	0.210	-2.1	189	0.230
	siRNA-eGFP	1	139	0.107	-2.9	144	0.115
10PE-DS-LNP	Patisiran	1	141	0.070	-3.5	145	0.081
	siRNA-eGFP	1	150	0.090	-1.8	159	0.112
LUVs	N/A	N/A	161	0.102	-30.3	158	0.089

Supplementary Table 3. IC_{50}-values of LNPs determined from eGFP silencing in U2OS and HeLa cell lines.

HeLa-CD63-eGFP				
	10PE-LNP-NP1	30PE-LNP-NP1	49PE-LNP-NP1	10PE-DS-LNP-NP1
IC50 (nM)	57.2	15.28	8.976	39.72
95% CI (profile likelihood)				
IC50 (nM)	52,88 to 62,36	14,50 to 16,09	8,297 to 9,684	37,08 to 42,60
**U2OS-UBE21-eGFP**				
	10PE-LNP-NP1	30PE-LNP-NP1	49PE-LNP-NP1	10PE-DS-LNP-NP1
IC50 (nM)	32.99	22.75	9.971	55.74
95% CI (profile likelihood)				
IC50 (nM)	31,39 to 34,72	21,34 to 24,27	8,900 to 11,08	51,38 to 60,94
**HeLa-CD63-eGFP**				
	- DiD + siGFP	+ DiD + siGFP		
IC50 (nM)	11.73	8.154		
95% CI (profile likelihood)				
IC50 (nM)	11.03 to 12.45	7.700 to 8.585		

Supplementary Table 4. Comparison of hexagonal and cubic model calculations based on Braggs peak maxima

The formulas for the calculation of d-spacings are described in other publications and were as follows:[1–3]

$$d_{hex} = \frac{2\pi n}{q}, with\, n = n[1,0] = 1, n[1,1] = \sqrt{3}$$
$$d_{cub} = \frac{2\pi n}{q}, with\, n = n[1,0] = \sqrt{2}, n[1,1] = \sqrt{3}$$

Sample	Structure	CryoEM value average (nm)	q_{max} value (Å)	Braggs peak	d-spacing SAXS hexagonal model (nm)	d-spacing SAXS cubic model (nm)
49PE-LNP-noRNA	Inverse hexagonal spheres	7.50 – 8.15	0.0749	**n[1,0]**	8.39	11.86
			0.127	**n[1,1]**	8.57	8.57
	Inverse hexagonal tubes	4.90	0.127	**n[1,0]**	4.95	6.99
49PE-LNP-NP1	Polymorphic	Not determined	0.0590	**n[1,0]**	10.65	15.0
			0.101	**n[1,1]**	10.78	10.78
	Inverse hexagonal tubes	6.30	0.101	**n[1,0]**	6.22	8.80

References

1. Demurtas, D. *et al.* Direct visualization of dispersed lipid bicontinuous cubic phases by cryo-electron tomography. *Nat. Commun.* **6**, 1–8 (2015).
2. Leung, S. S. W. & Leal, C. The stabilization of primitive bicontinuous cubic phases with tunable swelling over a wide composition range. *Soft Matter* **15**, 1269–1277 (2019).
3. Johnsson, M., Barauskas, J. & Tiberg, F. Cubic Phases and Cubic Phase Dispersions in a Phospholipid-Based System. *J. Am. Chem. Soc.* **127**, 1076–1077 (2005).
