# Supplementary material for: Liquid crystalline inverted lipid phases encapsulating siRNA enhance lipid nanoparticle mediated transfection
Source: Nat Commun. 2024 Feb 12;15:1303. doi: 10.1038/s41467-024-45666-5 (PMC10861598; doi:10.1038/s41467-024-45666-5)
Supplement: Supplementary file 3 — Reporting Summary [file 41467_2024_45666_MOESM3_ESM.pdf]

nature portfolio

Corresponding author(s): Thomas H. Sharp, Alexander Kros

Last updated by author(s): Nov 21, 2023

Reporting Summary

Nature Portfolio wishes to improve the reproducibility of the work that we publish. This form provides structure for consistency and transparency in reporting. For further information on Nature Portfolio policies, see our Editorial Policies and the Editorial Policy Checklist.

Statistics

For all statistical analyses, confirm that the following items are present in the figure legend, table legend, main text, or Methods section.

n/a	Confirmed	
☐	☒	The exact sample size (*n*) for each experimental group/condition, given as a discrete number and unit of measurement
☐	☒	A statement on whether measurements were taken from distinct samples or whether the same sample was measured repeatedly
☒	☐	The statistical test(s) used AND whether they are one- or two-sided<br>*Only common tests should be described solely by name; describe more complex techniques in the Methods section.*
☒	☐	A description of all covariates tested
☒	☐	A description of any assumptions or corrections, such as tests of normality and adjustment for multiple comparisons
☐	☒	A full description of the statistical parameters including central tendency (e.g. means) or other basic estimates (e.g. regression coefficient) AND variation (e.g. standard deviation) or associated estimates of uncertainty (e.g. confidence intervals)
☒	☐	For null hypothesis testing, the test statistic (e.g. *F*, *t*, *r*) with confidence intervals, effect sizes, degrees of freedom and *P* value noted<br>*Give P values as exact values whenever suitable.*
☒	☐	For Bayesian analysis, information on the choice of priors and Markov chain Monte Carlo settings
☒	☐	For hierarchical and complex designs, identification of the appropriate level for tests and full reporting of outcomes
☒	☐	Estimates of effect sizes (e.g. Cohen's *d*, Pearson's *r*), indicating how they were calculated

Our web collection on statistics for biologists contains articles on many of the points above.

Software and code

Policy information about availability of computer code

Data collection	CryoTEM images were collected using FEI software Tomography 4.0. Tilt series were collected using Tomo 4.0 (FEI)
Data analysis	Single particle cryoTEM analysis was performed using ImageJ. Tomograms were processed using IMOD software (v4.x), using fiducial tracking and reconstructed with both weighter back-projection and 5 iterations of a SIRT-like filter withing IMOD to enhance constrast. Tomograms were visualised and analysed using a combination of IMOD (v4.x) and UCSF Chimera (v1.17). DOPE and DODAP lipids were built using PyMOL (v2.x), before paramterization in eLBOW withing Phenix (v1.19). Lipids were relaxed into varied appropriate geometries using ISOLDE within UCSF ChimeraX (v1.17). UCSF Chimera (v1.17) was used to build models of siRNA and lipid-siRNA structures. Plotting of data and statistical analyses were performed using GraphPad Prism (v9.x or v11.x)

For manuscripts utilizing custom algorithms or software that are central to the research but not yet described in published literature, software must be made available to editors and reviewers. We strongly encourage code deposition in a community repository (e.g. GitHub). See the Nature Portfolio guidelines for submitting code & software for further information.

Data

Policy information about availability of data

All manuscripts must include a data availability statement. This statement should provide the following information, where applicable:

- Accession codes, unique identifiers, or web links for publicly available datasets
- A description of any restrictions on data availability
- For clinical datasets or third party data, please ensure that the statement adheres to our policy

CryoTEM micrographs from all the figures in the main text and supplementary information have been deposited within the public image database figshare.com (10.6084/m9.figshare.24456781). Source data are provided with this paper.

Human research participants

Policy information about studies involving human research participants and Sex and Gender in Research.

Reporting on sex and gender	Not applicable, this data has not been collected
Population characteristics	Not applicable, this data has not been collected
Recruitment	Not applicable, this data has not been collected
Ethics oversight	Not applicable, this data has not been collected

Note that full information on the approval of the study protocol must also be provided in the manuscript.

Field-specific reporting

Please select the one below that is the best fit for your research. If you are not sure, read the appropriate sections before making your selection.

☒ Life sciences ☐ Behavioural & social sciences ☐ Ecological, evolutionary & environmental sciences

For a reference copy of the document with all sections, see nature.com/documents/nr-reporting-summary-flat.pdf

Life sciences study design

All studies must disclose on these points even when the disclosure is negative.

Sample size	No sample-size calculations were performed. Sample size was determined to be adequate based on the observed standard deviations and statistical tests for different groups.
Data exclusions	No data were excluded from analysis.
Replication	All micrographs and scattering data were collected from at least two replicate experiments, the exact number is given per figure. Lipid mixing and cell culture experiments were carried out with at least three replicates. Attempts of data replication were successful.
Randomization	Samples were randomly allocated to corresponding experimental groups. Micrograph analysis was performed on random images from replicate experiments.
Blinding	Data collection and analysis were not formally blinded. The determinations performed in this article are considered to be an objective measure, supported by (semi) quantitative processing that is not subject to bias and therefor the integrity of the results are not impacted when running the study or analysis unblinded.

Reporting for specific materials, systems and methods

We require information from authors about some types of materials, experimental systems and methods used in many studies. Here, indicate whether each material, system or method listed is relevant to your study. If you are not sure if a list item applies to your research, read the appropriate section before selecting a response.

Materials & experimental systems

n/a	Involved in the study
☒	☐ Antibodies
☐	☒ Eukaryotic cell lines
☒	☐ Palaeontology and archaeology
☒	☐ Animals and other organisms
☒	☐ Clinical data
☒	☐ Dual use research of concern

Methods

n/a	Involved in the study
☒	☐ ChIP-seq
☒	☐ Flow cytometry
☒	☐ MRI-based neuroimaging

Eukaryotic cell lines

Policy information about cell lines and Sex and Gender in Research

Cell line source(s)	Cervical cancer (HeLa) and epithelial cells (U2OS) expressing eGFP-protein fusion. Cell lines were internally managed by Leiden University Medical Centre (Leiden, Netherlands)
Authentication	The cell line was not independently authenticated.
Mycoplasma contamination	All cell lines were routinely tested (negatively) for mycoplasma contamination
Commonly misidentified lines (See ICLAC register)	No commonly misidentified cell lines were used.
